# The potential role of serum amyloid A as biomarker of rheumatic diseases: a systematic review and meta-analysis

**DOI:** 10.1007/s10238-024-01413-0

**Published:** 2024-06-29

**Authors:** Angelo Zinellu, Arduino A. Mangoni

**Affiliations:** 1 Department of Biomedical Sciences, University of Sassari, Sassari, Italy; 2 Discipline of Clinical Pharmacology, College of Medicine and Public Health, Flinders University, Bedford Park, Adelaide, SA 5042 Australia; 3 Department of Clinical Pharmacology, Flinders Medical Centre, Southern Adelaide Local Health Network, Adelaide, Australia

**Keywords:** Serum amyloid A, Rheumatic diseases, Autoimmunity, Inflammation, Biomarkers, Active disease, Diagnostic accuracy

## Abstract

The identification of novel, robust biomarkers for the diagnosis of rheumatic diseases (RDs) and the presence of active disease might facilitate early treatment and the achievement of favourable long-term outcomes. We conducted a systematic review and meta-analysis of studies investigating the acute phase reactant, serum amyloid A (SAA), in RD patients and healthy controls to appraise its potential as diagnostic biomarker. We searched PubMed, Scopus, and Web of Science from inception to 10 April 2024 for relevant studies. We evaluated the risk of bias and the certainty of evidence using the JBI Critical Appraisal Checklist and GRADE, respectively (PROSPERO registration number: CRD42024537418). In 32 studies selected for analysis, SAA concentrations were significantly higher in RD patients compared to controls (SMD = 1.61, 95% CI 1.24–1.98, *p* < 0.001) and in RD patients with active disease compared to those in remission (SMD = 2.17, 95% CI 1.21–3.13, *p* < 0.001). Summary receiving characteristics curve analysis showed a good diagnostic accuracy of SAA for the presence of RDs (area under the curve = 0.81, 95% CI 0.78–0.84). The effect size of the differences in SAA concentrations between RD patients and controls was significantly associated with sex, body mass index, type of RD, and study country. Pending the conduct of prospective studies in different types of RDs, the results of this systematic review and meta-analysis suggest that SAA is a promising biomarker for the diagnosis of RDs and active disease.

**Supplementary Information:**

The online version contains supplementary material available at 10.1007/s10238-024-01413-0.

## Introduction

Clinicians often face significant challenges with diagnosing a range of autoimmune (e.g., systemic sclerosis, SSc, rheumatoid arthritis, RA, and systemic lupus erythematosus, SLE), mixed-autoimmune-autoinflammatory (e.g., ankylosing spondylitis, AS, psoriatic arthritis, PsA, and Behcet’s disease, BD), and autoinflammatory (e.g., familial Mediterranean fever, FMF) conditions, also comprehensively defined as “rheumatic diseases (RDs) [1, 2]. Such challenges are particularly vexing when physicians are confronted with mild, non-specific clinical manifestations, dubious imaging findings, and borderline results with serological markers [3–5]. At the same time, there is good evidence that early recognition and treatment is associated with favourable long-term outcomes in several types of RDs [6–9].

The typical dysregulation of inflammatory pathways in RDs, with consequent excess local and systemic inflammation, has led to the routine measurement of biomarkers of inflammation, e.g., C-reactive protein (CRP), erythrocyte sedimentation rate (ESR), for initial assessment and monitoring, in combination with clinical evaluation and serological biomarkers specific for individual RDs [10–12]. However, their limited diagnostic accuracy in observational studies has stimulated the search for novel, more robust biomarkers for diagnosing RDs and detecting changes in disease activity [13–16].

One potential candidate biomarker of RDs is represented by serum amyloid A (SAA) proteins. SAA proteins, primarily synthesized in the liver, are significantly activated during the acute phase response in the presence of inflammation [17]. Circulating SAA concentrations can increase up to 1000-fold within the first 24–48 h of an acute phase response because of several stimulating factors, primarily pro-inflammatory cytokines [18, 19]. SAA can in turn activate the complement system, the nucleotide-binding domain leucine-rich repeat-containing family pyrin-domain containing 3 inflammasome, and several pro-inflammatory cytokines [20–22]. Notably, in serum SAA is primarily bound to high density lipoprotein (HDL)-cholesterol, reducing the physiological anti-inflammatory effects of this lipoprotein [23]. A number of studies have also reported that SAA is involved in cholesterol transport and recycling and exerts significant pro-atherogenic effects [24–26]. Such effects may play a role in the complex interplay between dysregulated immunity, inflammation, and cardiovascular disease in RD patients [27, 28].

Given the potential pathophysiological role of SAA in RDs, we conducted a systematic review and meta-analysis of studies investigating this acute phase reactant in patients with RDs and healthy controls and in RD patients with and without active disease. We speculated that higher SAA concentrations were significantly associated with the presence of RDs and active disease. Where possible, we also investigated associations between the effect size of the between-group differences and several study and patient characteristics, including lipid profile and conventional inflammatory biomarkers, and the diagnostic accuracy of the SAA.

## Methods

### Study selection

We conducted a systematic search in the electronic databases PubMed, Web of Science, and Scopus from inception to 10 April 2024 using the following terms: “serum amyloid A” AND “rheumatic diseases” OR “rheumatoid arthritis” OR “psoriatic arthritis” OR “ reactive arthritis” OR “ankylosing spondylitis” OR “systemic lupus erythematosus” OR “systemic sclerosis” OR “scleroderma” OR “Sjogren’s syndrome” OR “connective tissue diseases” OR “vasculitis” OR “Behçet’s disease” OR “idiopathic inflammatory myositis” OR “polymyositis” OR “dermatomyositis” OR “gout” OR “pseudogout” OR”systemic vasculitis” OR “ANCA-associated vasculitis” OR “Takayasu arteritis” OR “polyarteritis nodosa” OR “osteoarthritis” OR “fibromyalgia” OR “granulomatous polyangiitis” OR”Henoch-Schonlein purpura” OR “granulomatous polyangiitis” OR “Wegener’s granulomatosis” OR “familial Mediterranean fever” OR “polymyalgia rheumatica” OR “temporal arteritis” OR “giant cell arteritis”.

Two investigators independently screened abstracts and full articles according to pre-defined inclusion and exclusion criteria. Inclusion criteria were: (i) the assessment of SAA concentrations, (ii) the comparison of patients with RDs and healthy controls in case–control studies, (iii) the inclusion of patients aged ≥ 18 years, (iv) the use of English language, and (v) the availability of the full-text of the article. Exclusion criteria were: (i) articles reporting duplicate or irrelevant data, (ii) the inclusion of participants under 18 years, and (iii) non-case–control studies. The investigators also hand searched the references of individual articles to identify additional studies.

The following variables were independently extracted for further analysis: year of publication, first author, country where the study was conducted, RD type and duration, sample size, age, male to female ratio, SAA concentrations, body mass index, C-reactive protein (CRP), erythrocyte sedimentation rate (ESR), total, LDL-, and HDL-cholesterol, triglycerides, use of disease-modifying antirheumatic drugs or corticosteroids, area under the receiver operating characteristic curve (AUROC) with 95% confidence intervals (CIs), sensitivity, specificity, and cut-off values used for SAA.

The Joanna Briggs Institute Critical Appraisal Checklist for analytical studies was used to assess the risk of bias of individual studies [29]. The Grades of Recommendation, Assessment, Development and Evaluation (GRADE) Working Group system were used to rank the certainty of evidence [30]. The study adhered to the Preferred Reporting Items for Systematic reviews and Meta-Analyses (PRISMA) 2020 statement (Supplementary Table 1) [31], and was registered in an international repository (PROSPERO registration number: CRD42024537418).

### Statistical analysis

We generated forest plots of standardized mean differences (SMDs) and 95% confidence intervals (CIs) to investigate differences in SAA concentrations between RD patients and healthy controls and between RD patients with active disease and those in remission (*p* < 0.05 for statistical significance). The Graph Data Extractor software was used to extract medians and interquartile ranges (San Diego, CA, USA). Established methods were used to extrapolate means and standard deviations from medians and interquartile ranges or ranges [32]. Heterogeneity was assessed using the Q statistic (*p* < 0.10 for statistical significance) [33, 34]. Sensitivity analysis was conducted to investigate the stability of the results of the meta-analysis [35]. Established methods were used to investigate the presence of publication bias [36–38]. Univariate meta-regression and subgroup analyses were conducted to investigate associations between the effect size and year of publication, study country, RD type and duration, sample size, age, male to female ratio, body mass index, CRP, ESR, total, LDL-, and HDL-cholesterol, triglycerides, and use of DMARDs and corticosteroids.

The diagnostic accuracy of SAA was assessed by calculating the pooled sensitivity and specificity and generating a forest plot [39]. Summary receiving characteristics (SROC) curve with 95% confidence region and prediction region was generated using the midas command in Stata [39]. The relationship between prior probability, likelihood ratio, and posterior test probability was assessed by Fagan’s nomogram plot [40]. All analyses were performed using Stata 14 (Stata Corp., College Station, TX, USA).

## Results

### Study selection

**Fig. 1:**
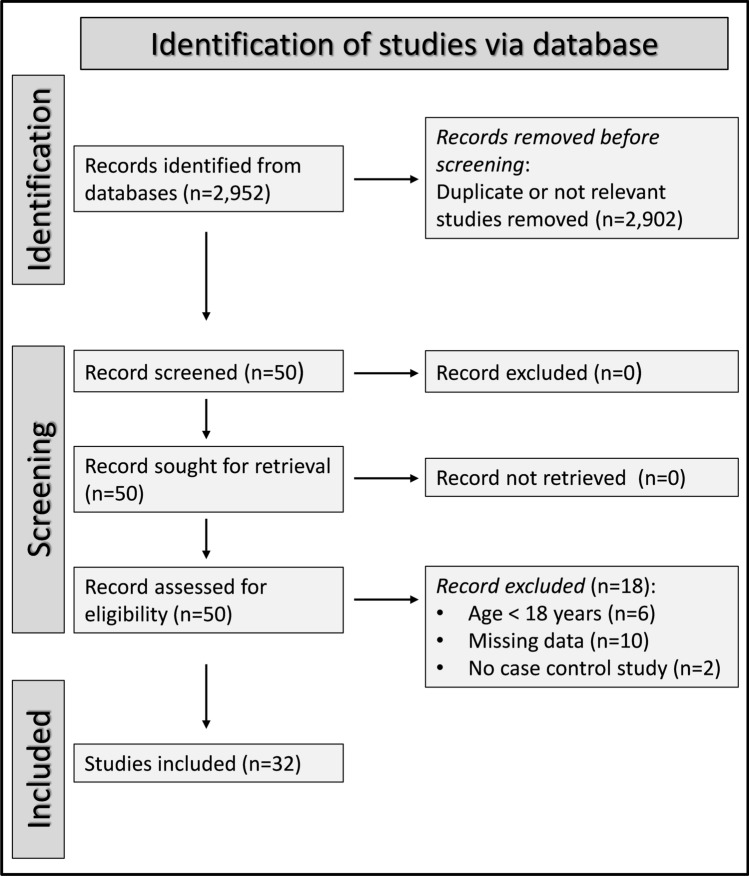
PRISMA 2020 flow diagram

A flow chart describing the screening and selection process is presented in Fig. 1. We initially identified 2952 articles, of which 2902 were immediately excluded because they presented duplicate or irrelevant data. After a full-text review of the remaining 50 articles, a further ten were excluded because of missing information, six because participants were younger than 18 years, and two because they were not a case–control study. This led to the selection of 32 studies for further analysis [41–72] (Tables 1, 2 and 3). The initial level of certainty was ranked as low given the cross-sectional design of the selected studies.

**Table 1:** Characteristics of the studies reporting serum amyloid a concentrations in patients with rheumatic diseases and healthy controls

References	Healthy controls				Patients with rheumatic diseases				Disease type
	n	Age (Years)	M/F	SAA (Mean ± SD)	n	Age (Years)	M/F	SAA (Mean ± SD)	
Aygündüz et al. [41], Turkey	27	37.1	14/13	50 ± 23	43	34.2	22/21	220 ± 51	BD
Tanimoto et al. [42], Japan	25	61	2/23	9 ± 9	25	61	2/23	63 ± 122	RA
Wong et al.,[43] Australia	53	54.7	Matched	4.8 ± 3.7	53	55	Matched	19.8 ± 19.2	RA
Jung et al. [44], Korea	38	30.1	25/13	2.73 ± 1.57	38	29.6	26/12	9.52 ± 7.49	AS
Karadag et al. [45], Turkey	22	38	0/22	19.6 ± 7.5	25	40	0/25	38.8 ± 24	SLE
Sato et al. (a) [46], Japan	17	43.4	0/17	2.68 ± 0.44	14	42.3	0/14	12.3 ± 9	SLE
Sato et al. (b) [46], Japan	22	57.9	0/22	3.63 ± 1.47	25	56.8	0/25	10.7 ± 8.7	SLE
Rho et al. [47], USA	92	53.2	34/58	1.83 ± 1.35	169	54.2	52/117	5.3 ± 7.1	RA
Ma et al. [48], China	20	27.45	2/18	23.9 ± 37.1	43	28.3	5/38	76.4 ± 47.7	TA
Rooney et al.,[49] USA	62	48	31/31	3.53 ± 3.42	105	51.7	17/88	7.4 ± 7.0	RA
He et al. [50], China	20	Matched	Matched	2.68 ± 1.56	70	Matched	Matched	68.72 ± 48.77	HSP
Londono et al. [51], Colombia	46	NR	NR	282.49 ± 371.94	62	31.9	43/19	853.7 ± 946.2	SpA
de Seny et al. (a) [52], Belgium	35	56.7	15/20	1.31 ± 0.44*	29	Matched	Matched	1.57 ± 0.29*	OA
de Seny et al. (b) [52], Belgium	35	56.7	15/20	1.31 ± 0.44*	27	Matched	Matched	2.42 ± 0.91*	RA
Giese et al. [53], Germany	37	33.7	17/20	0.37 ± 0.26	30	33.5	14/16	3.14 ± 4.82	FMF
Lakota et al. [54], Slovenia	98	43.39	64/34	7.29 ± 19.17	129	52.28	20/109	32.39 ± 85.89	SSc
Shen et al. (a) [55], China	50	52	12/38	1.45 ± 0.72	88	58	23/65	6.15 ± 3.27	RA
Shen et al. (b) [55], China	50	52	12/38	1.45 ± 0.72	43	53	12/31	2.54 ± 0.31	SLE
Shen et al. (c) [55], China	50	52	12/38	1.45 ± 0.72	54	56	12/42	1.42 ± 0.97	OA
Botta et al. [56], Argentina	33	49	7/26	6.7 ± 5.5	44	56	5/39	8.0 ± 6.5	RA
Ciftci et al. [57], Turkey	37	36.8	7/30	1154.78 ± 1375.15	50	36.6	9/41	2126.5 ± 1876.34	FMF
Gaál K et al. [58], Hungary	49	31.8	8/41	2666.05 ± 2301.11	51	31.82	7/44	19,017.07 ± 25,899.25	SLE
Uslu AU et al. [59], Turkey	40	32.4	12/28	1.68 ± 0.63	40	30.2	11/29	25.2 ± 45.78	FMF
Fentoglu et al. [60], Turkey	128	31.91	54/74	2.82 ± 0.23	109	32.65	47/62	18.51 ± 1.17	FMF
Nair et al. [61], India	40	29	12/28	96.6 ± 66.7	99	31	23/76	140 ± 69.8	TA
Lis-Swiety et al. [62], Poland	11	NR	Matched	18.97 ± 18.57	33	49.7	9/24	62.3 ± 23.2	SSc
Bezuidenhout et al. [63], South Africa	25	53.4	8/17	2.37 ± 3.72	30	53.4	6/24	8.6 ± 12.8	RA
van Sleen et al. [64], The Netherlands	33	67	11/22	5.67 ± 10.2	29	70	11/18	197 ± 397	PMR
Yuan et al. (a) [65], China	25	48.56	5/20	4.9 ± 1.96	25	48.52	5/20	117.64 ± 107.37	RA
Yuan et al. (b) [65], China	25	48.52	5/20	4.25 ± 1.16	25	48.96	5/20	50.74 ± 74.16	RA
Yuan et al. (c)[65], China	25	48.28	5/20	5.16 ± 2.61	25	48.32	5/20	67.82 ± 91.59	RA
Can Sandikci et al. [66], Turkey	28	Matched	16/12	1.81 ± 1.81	54	35.78	27/27	9.21 ± 16.85	FMF
Ciregia et al. [67], India	100	34.4	20/80	4.22 ± 0.49*	100	34.5	20/80	4.64 ± 0.71*	RA
Hu et al. [68], China	80	31.33	51/29	7.64 ± 1.32	78	36.24	50/28	39.65 ± 14.23	AS
Luo et al.[69], China	40	23.58	2/38	54.6 ± 18.8	80	23.73	4/76	91.7 ± 24.29	TA
Sweet et al.[70], USA	34	46.6	16/18	21.1 ± 1.15*	300	47.5	154/146	22.89 ± 2.28*	PsA
El Kosaier et al. [71], Egypt	60	45.9	13/47	1.6 ± 0.12	60	45.6	11/49	35.1 ± 3.6	RA
Zhou et al. [72], China	20	35.75	NR	2.13 ± 0.89	61	41.4	NR	56.7 ± 56.7	Gout

*AS*, ankylosing spondylitis; *BD*, Behcet’s disease; *FMF*, familial Mediterranean fever; *HSP*, Henoch–Schönlein purpura; *M/F*, male to female ratio; *OA*, osteoarthritis; *PMR*, polymyalgia rheumatica; *PsA*, psoriatic arthritis; *RA*, rheumatoid arthritis; *SAA*, serum amyloid A; *SLE*, systemic lupus erythematosus; *SpA*, spondylarthritis; *SSc*, systemic sclerosis; *TA*, Takayasu arteritis; *, log-transformed data

SAA concentrations reported as mg/L or µg/L

**Table 2:** Characteristics of the studies investigating the diagnostic accuracy of serum amyloid A concentrations for rheumatic diseases

Study	Disease	N	Age (years)	M/F	AUC (95% CI)	Sensitivity (%)	Specificity (%)
Lakota et al. [54], Slovenia	SSc	227	48.4	84/143	0.73 (0.667–0.786)	0.6589	0.7551
Ciftci et al. [57], Turkey	FMF	87	36.7	16/71	0.719 (NR)	0.82	0.568
Uslu et al. [59] Turkey	FMF	80	31.3	23/57	0.677 (0.560–0.782)	0.625	0.6
Bezuidenhout et al. [63] South Africa	RA	55	53.4	14/41	0.799 (0.669–0.895)	0.7333	0.8
Yuan et al. (a) [65] China	RA	50	48.5	10/40	0.99 (NR)	0.96	0.92
Yuan et al. (b) [65] China	RA	50	48.7	10/40	0.65 (NR)	0.52	1
Yuan et al. (c) [65] China	RA	50	48.3	10/40	0.82 (NR)	0.72	0.92
Ciregia et al. [67] India	RA	200	34.5	40/160	0.698 (NR)	0.6	0.678
Hu et al. [68] China	AS	158	33.8	101/57	NR (NR)	0.7949	0.775

*AS*, ankylosing spondylitis; *AUC*, area under the curve; *CI*, confidence interval; *FMF*, familial Mediterranean fever; *M/F*, male to female ratio; *NR*, not reported; *RA*, rheumatoid arthritis; *SSc*, systemic sclerosis

**Table 3:** Characteristics of studies reporting serum amyloid A concentrations in patients with rheumatic diseases with active disease and remission

Study	Patients in remission				Patients with active disease				Disease type
	n	Age (Years)	M/F	SAA (Mean ± SD)	n	Age (Years)	M/F	SAA (Mean ± SD)	
Aygündüz et al. Turkey [41]	23	35.4	8/15	153 ± 41	20	33.1	14/6	296 ± 92	BD
Ma et al. [48] China	25	30.56	3/22	49.2 ± 60.7	18	25.17	2/16	95.9 ± 38.4	TA
Botta et al. [56]Argentina	17	56	3/14	6.8 ± 5.87	27	56	2/25	8.7 ± 6.8	RA
Nair et al. [61]India	48	33.1	9/39	103 ± 45	43	28.7	14/29	175 ± 64	TA
Hu et al. [68]China	36	NR	NR	20.36 ± 5.36	42	NR	NR	56.18 ± 17.25	AS
Luo et al. [69]China	40	23.7	2/38	78.7 ± 23.3	40	23.8	2/38	104.7 ± 25.28	TA
El Kosaier et al. [71]Egypt	10	NR	NR	1.4 ± 0.16	50	NR	NR	41.8 ± 2.9	RA
Zhou et al. [72]China	27	39.67	NR	5.73 ± 5.43	34	42.82	NR	97.25 ± 164.8	Gout

*AS*, ankylosing spondylitis; *BD*: behcet’s disease; *M/F*, male to female ratio; *NR*: not reported; *RA*: rheumatoid arthritis; *SAA*, serum amyloid A; *TA*: Takayasu arteritis

### Serum amyloid A and rheumatic diseases

The association between SAA and RDs was reported in 32 studies (38 group comparators) evaluating a total of 2365 RD patients (mean age 44 years, 69% females) and 1632 healthy controls (mean age 43 years, 66% females) [41–72] (Table 1). Eighteen studies were conducted in Asia [41, 42, 44–46, 48, 50, 55, 57, 59–61, 65–69, 72], six in Europe [52–54, 58, 62, 64], five in America [47, 49, 51, 56, 70], two in Africa [63, 71], and one in Oceania [43]. Thirteen study comparators included patients with RA [42, 43, 47, 49, 52, 55, 56, 63, 65, 67, 71], five with SLE [45, 46, 55, 58], five with FMF [53, 57, 59, 60, 66], three with Takayasu arteritis (TA) [48, 61, 69], two with AS [44, 68], two with SSc [54, 62], two with osteoarthritis (OA) [52, 55], one with BD [41], one with Henoch–Schönlein purpura (HSP) [50], one with spondylarthritis (SpA) [51], one with polymyalgia rheumatica (PMR) [64], one with PsA [70], and one with gout [72]. Mean RD duration was reported in 14 studies and ranged between 2.7 and 21.7 years [43–46, 52, 59, 61–64, 66, 70, 71].

The risk of bias was considered low in 25 studies [42–50, 52, 56–63, 65, 66, 68–72] and moderate in the remaining seven [41, 51, 53–55, 64, 67] (Supplementary Table 2).

**Fig. 2:**
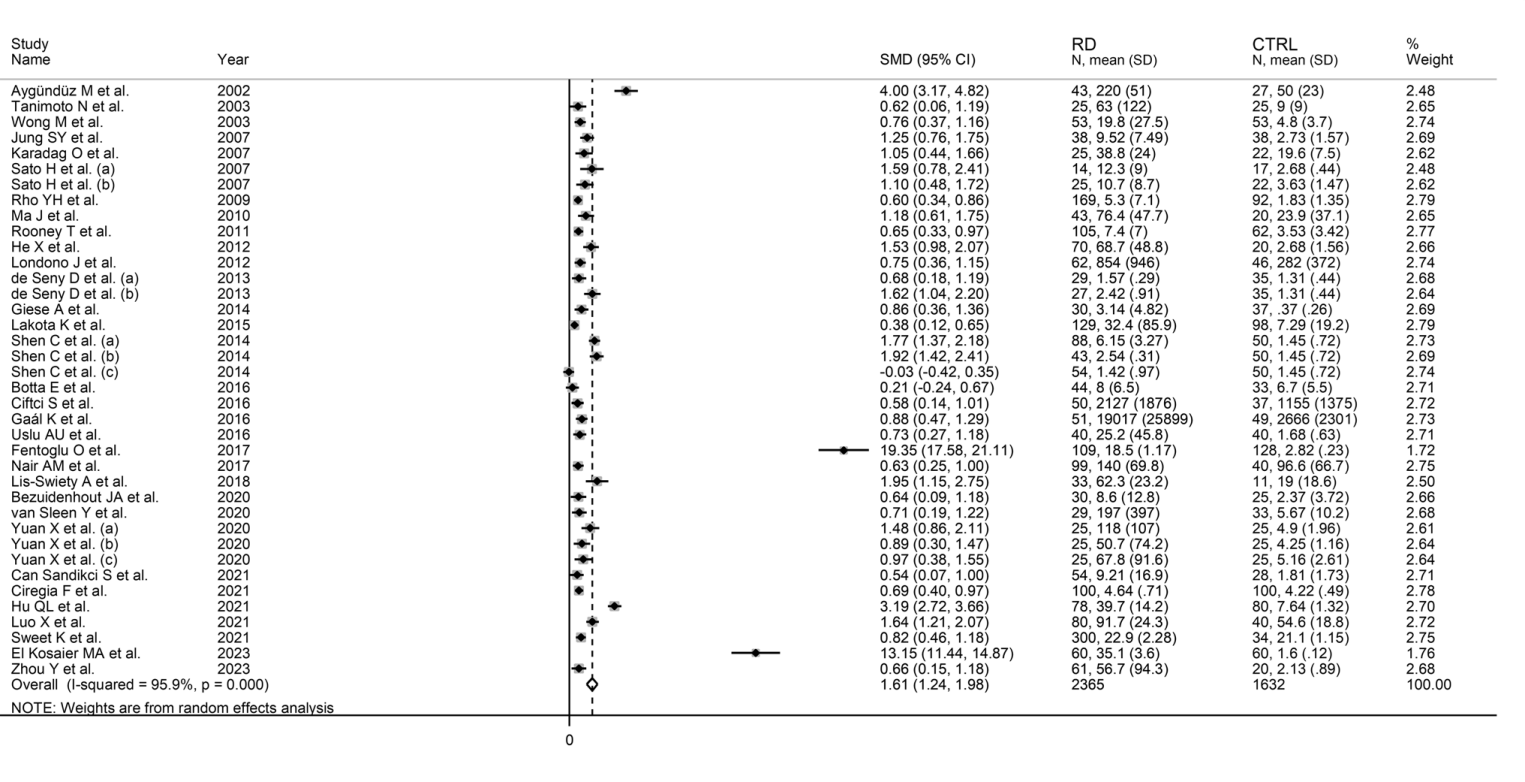
Forest plot of studies reporting serum amyloid A concentrations in patients with rheumatic diseases and healthy controls

**Fig. 3:**
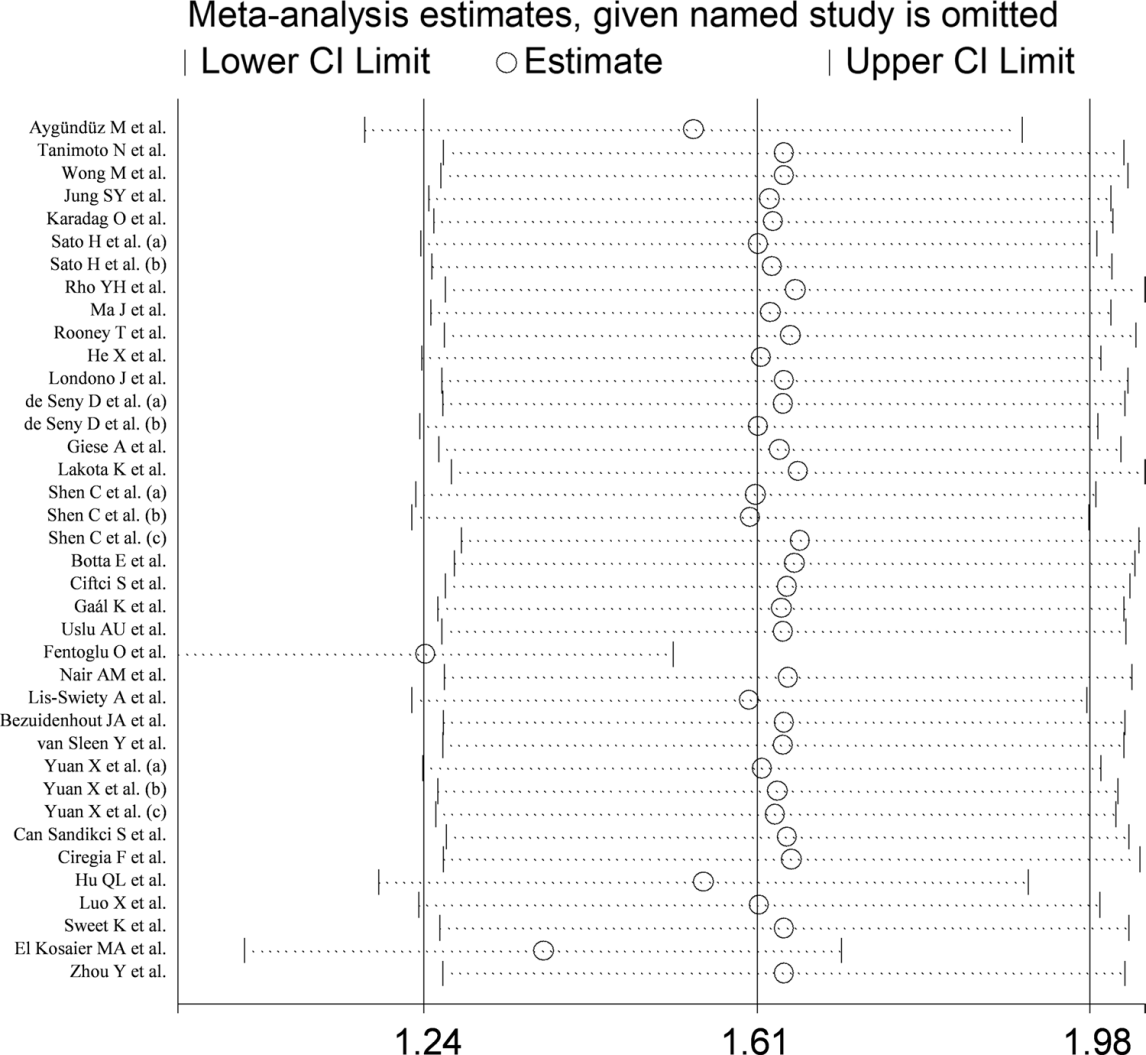
Sensitivity analysis of the association between serum amyloid A concentrations and rheumatic diseases

**Fig. 4:**
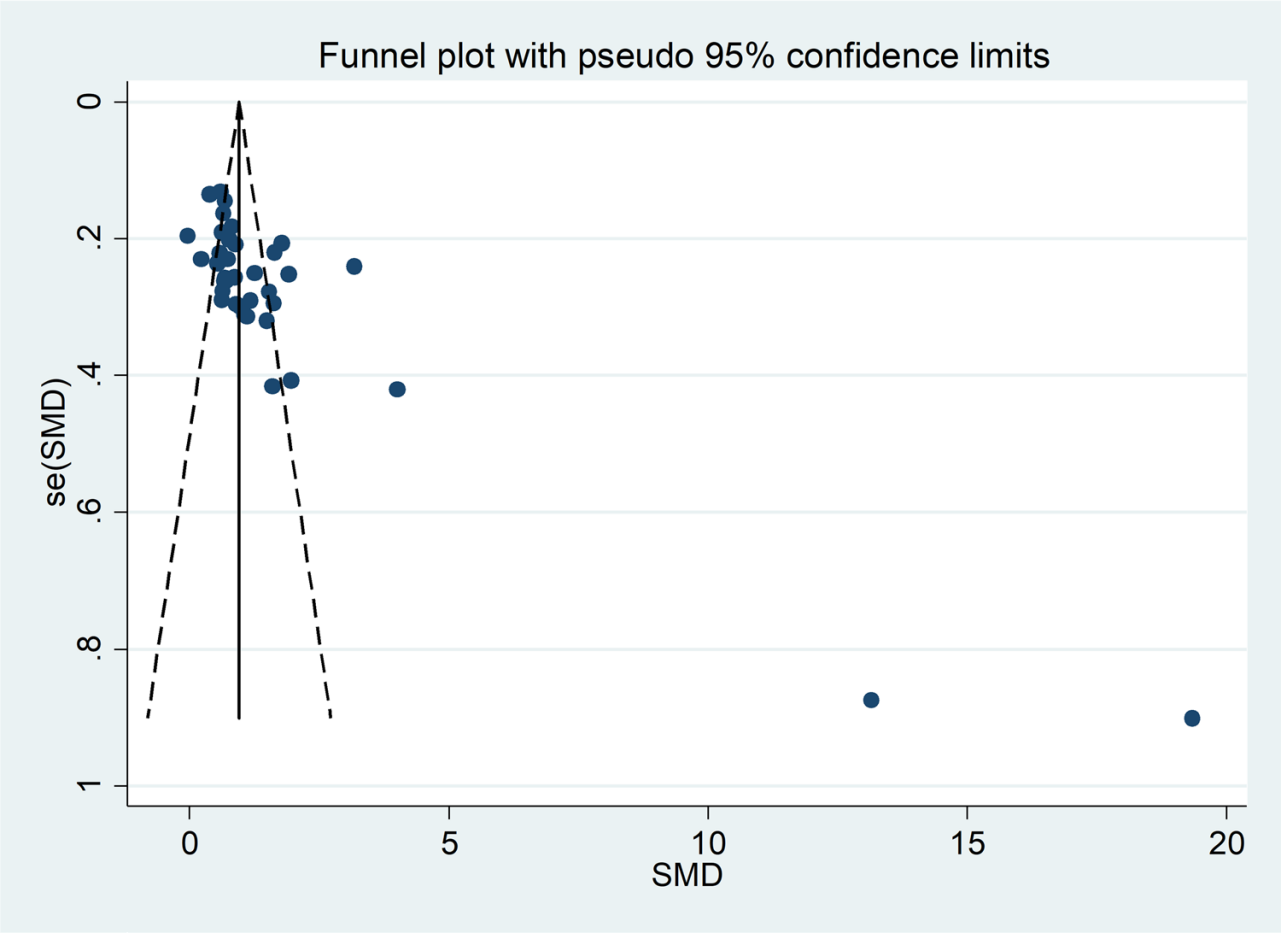
Funnel plot of studies investigating the association between serum amyloid A concentrations and rheumatic diseases

The forest plot showed that the concentrations of SAA were overall significantly higher in RD patients when compared to controls (SMD = 1.61, 95% CI 1.24–1.98, *p* < 0.001; I^2^ = 95.9%, *p* < 0.001; Fig. 2). Sensitivity analysis revealed that two studies showed a significant effect on the results of the meta-analysis of two studies [60, 71] (the effect size ranged between 1.24 and 1.66, Fig. 3). This finding was further corroborated by funnel plot analysis, which revealed a marked distortive effect in the symmetry graph attributable to these studies (Fig. 4). Their removal led to a reduction in the pooled SMD which, however, remained significant (SMD = 1.06, 95% CI 0.85–1.28, *p* < 0.001, I^2^ = 87.7%, *p* < 0.001).

**Fig. 5:**
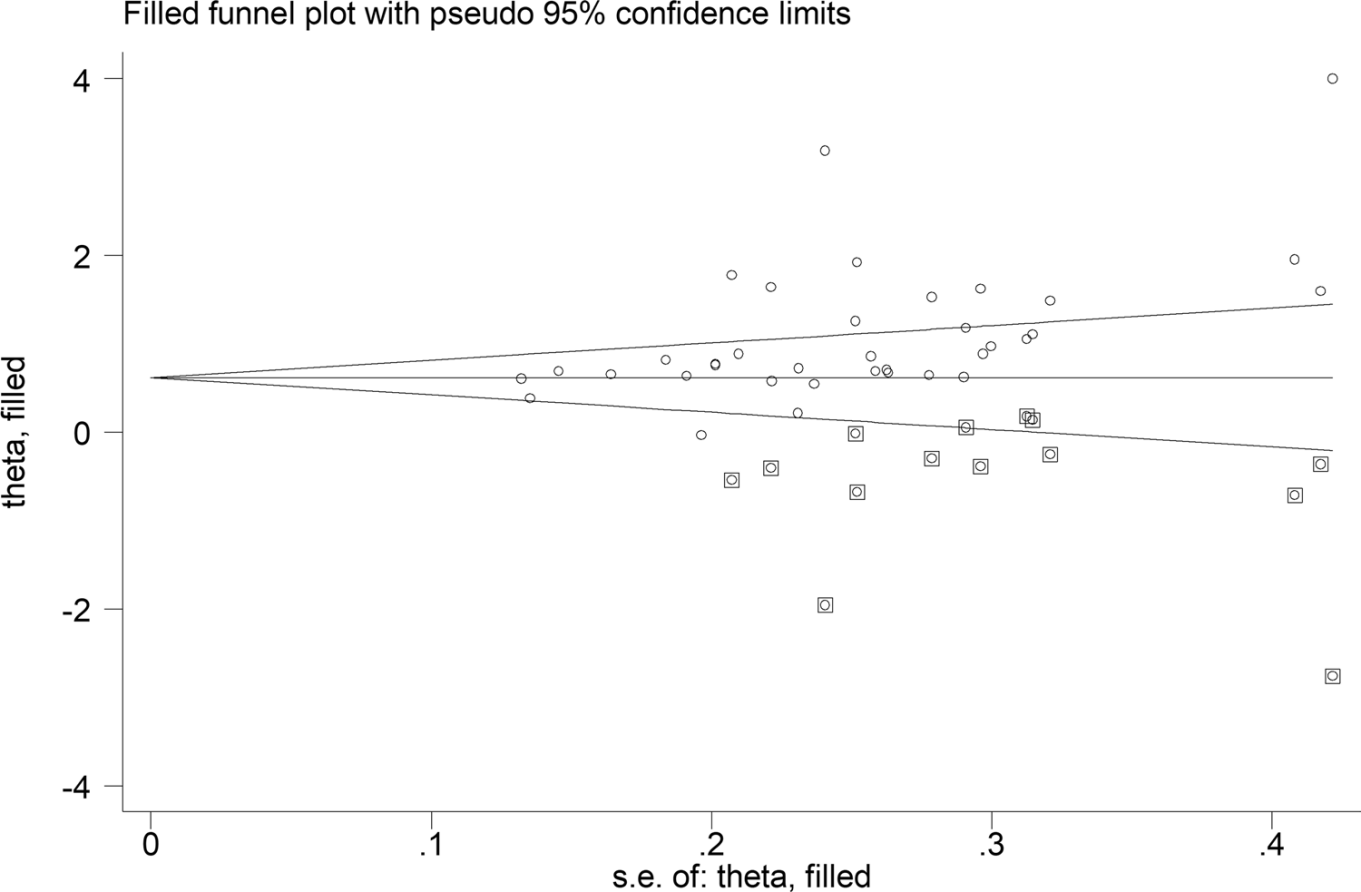
Funnel plot of studies investigating the association between serum amyloid A concentrations and rheumatic diseases after “trimming and filling”. Dummy studies and genuine studies are represented by enclosed circles and free circles, respectively

There was significant publication bias (Begg’s test, *p* = 0.001; Egger’s test, *p* = 0.002), which remained after removing the aforementioned studies [60, 71]. The “trim-and-fill” method identified 14 missing studies to be added to the left part of funnel plot to ensure symmetry (Fig. 5). The resulting pooled SMD was further reduced yet still significant (SMD = 0.63, 95% CI 0.38 to 1.87, *p* < 0.001).

**Fig. 6:**
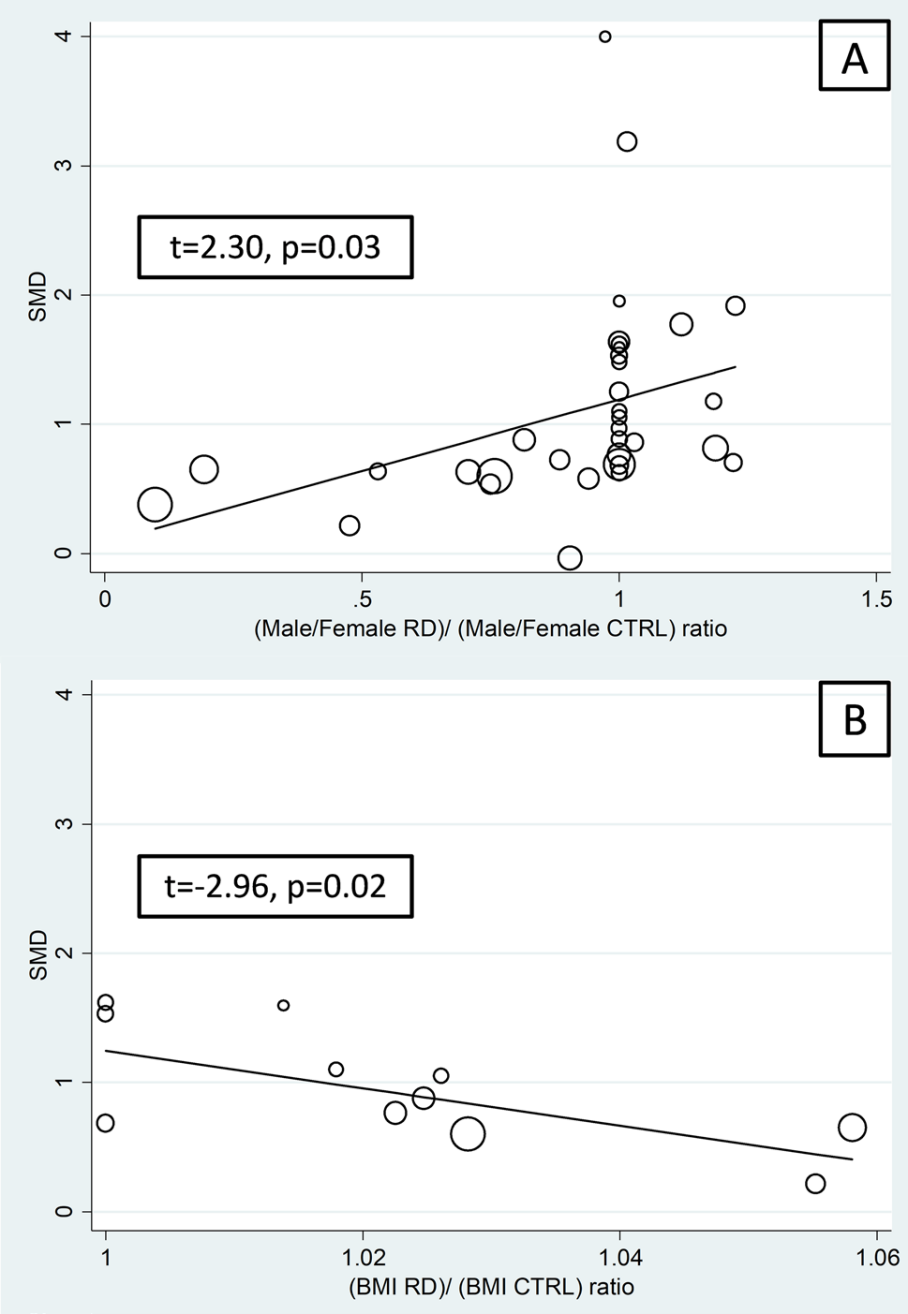
Bubble plot reporting the univariate meta-regression analysis between the effect size and male to female ratio (A) and body mass index (B)

Univariate meta-regression analysis revealed the absence of significant associations between the effect size and the between-group differences in SAA concentrations and age (t = −0.72, *p* = 0.47), sample size (t =  −0.79, *p* = 0.44), RD duration (t = 0.19, *p* = 0.85), CRP (t =  − 0.64, *p* = 0.53), ESR (t = 0.08, *p* = 0.93), total (t = 1.36, *p* = 0.21) and LDL-cholesterol (t = 1.69, *p* = 0.13), and use of DMARDs (t =  − 1.56, *p* = 0.14) or corticosteroids (t = 0.29, *p* = 0.78). By contrast, significant associations were observed between the effect size and male to female ratio (t = 2.30, *p* = 0.03) and body mass index (t =  − 2.96, *p* = 0.02; Fig. 6), with a non-significant trend for HDL-cholesterol (t = 2.01, *p* = 0.08).

**Fig. 7:**
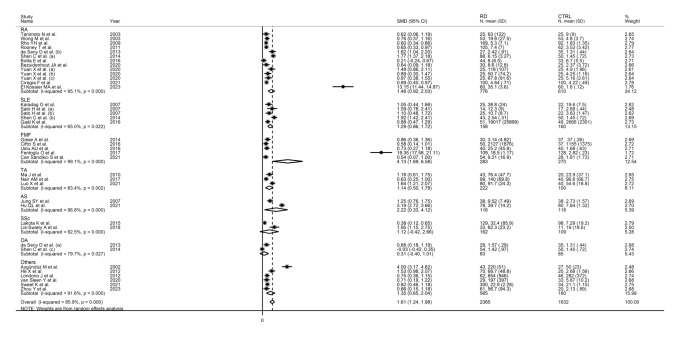
Forest plot of studies reporting serum amyloid A concentrations in patients with rheumatic diseases and healthy controls according to disease type

**Fig. 8:**
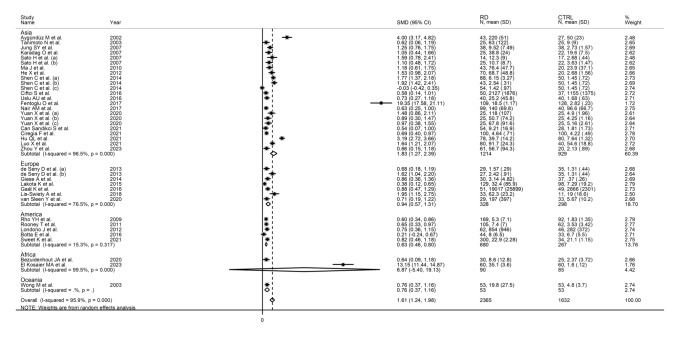
Forest plot of studies reporting serum amyloid A concentrations in patients with rheumatic diseases and healthy controls according to geographical area

Subgroup analysis showed that the pooled SMD was statistically significant in studies of RA (SMD = 1.48, 95% CI 0.92–2.03, *p* < 0.001; I^2^ = 95.1%, *p* < 0.001), SLE (SMD = 1.29, 95% CI 0.86–1.72, *p* < 0.001; I^2^ = 65.0%, *p* = 0.02), FMF (SMD = 4.13, 95% CI 1.69–6.58, *p* < 0.001; I^2^ = 99.1%, *p* < 0.001), TA (SMD = 1.14, 95% CI 0.50 to 1.78, *p* = 0.001; I^2^ = 83.4%, *p* = 0.002) and AS patients (SMD = 2.22, 95% CI 0.33–4.12, *p* = 0.021; I^2^ = 96.8%, *p* < 0.001), but not SSc (SMD = 1.12, 95% CI − 0.42–2.66, *p* = 0.15; I^2^ = 92.5%, *p* < 0.001) or OA patients (SMD = 0.31, 95% CI − 0.40–1.01, *p* = 0.40; I^2^ = 79.7%, *p* = 0.027; Fig. 7), with a relatively lower heterogeneity in the SLE subgroup (I^2^ = 65.0%). Furthermore, the pooled SMD was significant in studies conducted in Asia (SMD = 1.83, 95% CI 1.27 to 2.39, *p* < 0.001; I^2^ = 96.5%, *p* < 0.001), Europe (SMD = 0.94, 95% CI 0.57 to 1.31, *p* < 0.001; I^2^ = 76.5%, *p* < 0.001), and America (SMD = 0.63, 95% CI 0.46 to 0.80, *p* < 0.001; I^2^ = 15.3%, *p* = 0.317), but not Africa (SMD = 6.87, 95% CI − 5.40 to 19.13, *p* = 0.45; I^2^ = 99.5%,* p* < 0.001; Fig. 8), with a relatively low between-study variance in the American subgroup (I^2^ = 15.3%).

The overall level of certainty was upgraded to moderate after considering the low-moderate risk of bias in all studies (no change), the high but partially explainable heterogeneity (no change), the lack of indirectness (no change), the large effect size (SMD = 1.61, upgrade one level) [73], and the presence of publication bias which was partially addressed using the “trim-and-fill” method (no change).

### Diagnostic accuracy of serum amyloid A for the presence of rheumatic diseases

Five studies reported the ROC analysis of the diagnostic accuracy of SAA concentrations for RDs [57, 59, 65, 67, 68]. A de novo ROC analysis was conducted using data from two additional studies [54, 63]. Sensitivity and specificity were extracted from these seven studies (nine comparator groups) which investigated a total of 910 participants (649 RD patients and 308 healthy controls, 68% females, mean age 41 years) (Table 2). Five studies were conducted in Asia [57, 59, 65, 67, 68], one in Europe [54], and one in Africa [63]. Five comparator groups included individuals with RA [63, 65, 67], two with FMF [57, 59], one with SSc [54], and one with AS [68].

The risk of bias was assessed as low in five studies [57, 59, 63, 65, 68], and moderate in the remaining two [54, 67] (Supplementary Table 2).

**Fig. 9:**
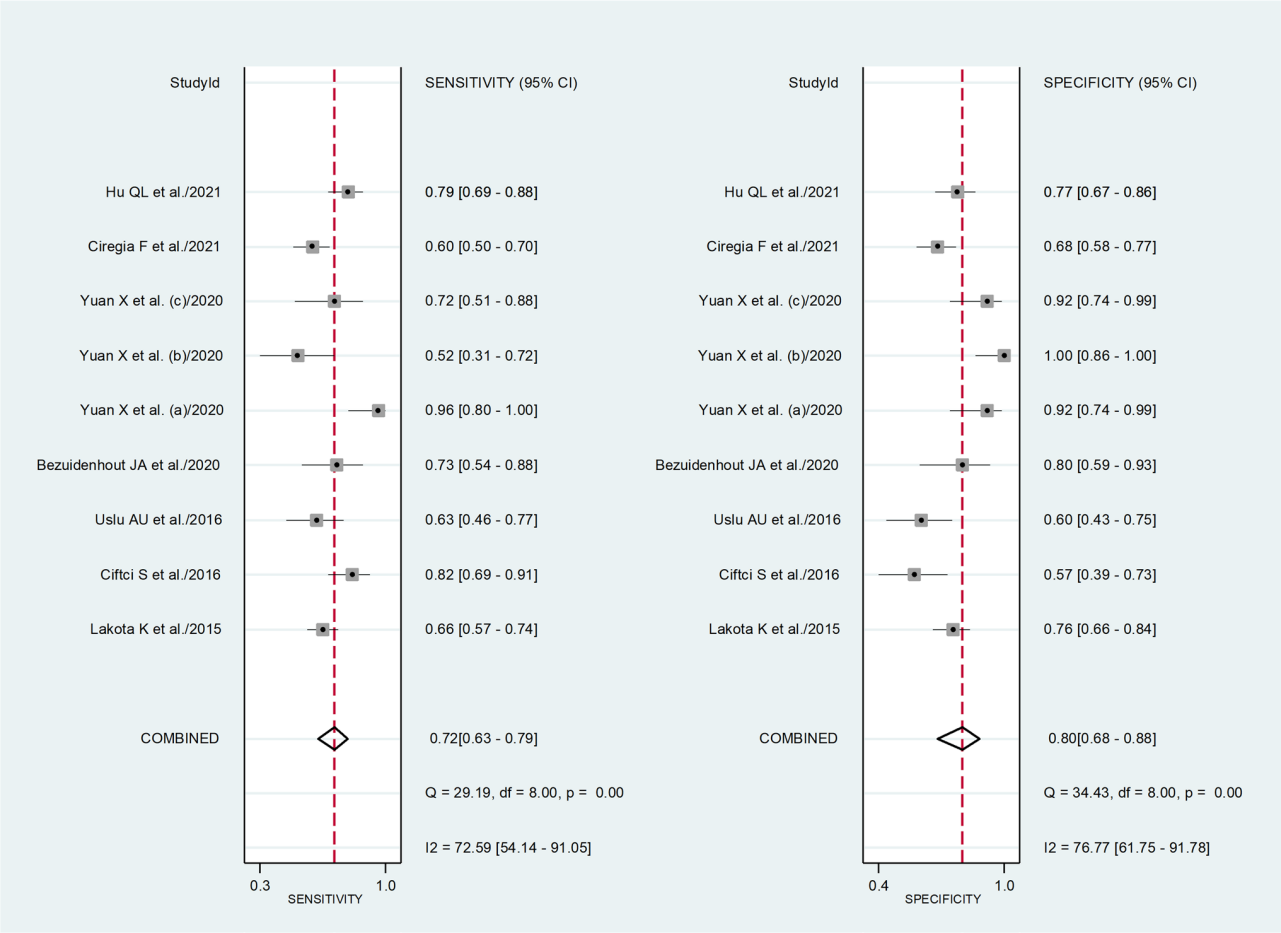
Forest plot of the pooled estimates of sensitivity and specificity of serum amyloid A concentrations for the presence of rheumatic diseases

**Fig. 10:**
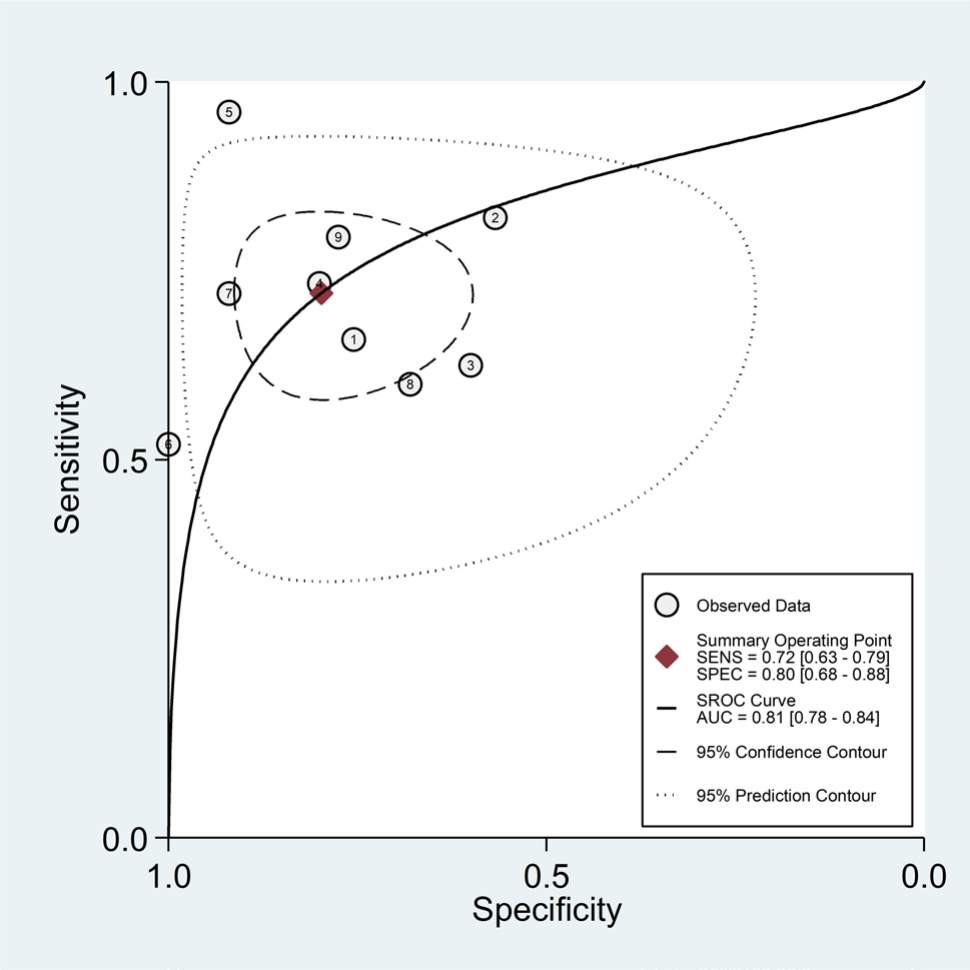
SROC curve with 95% confidence region and prediction region of serum amyloid A concentrations for the presence of rheumatic diseases

**Fig. 11:**
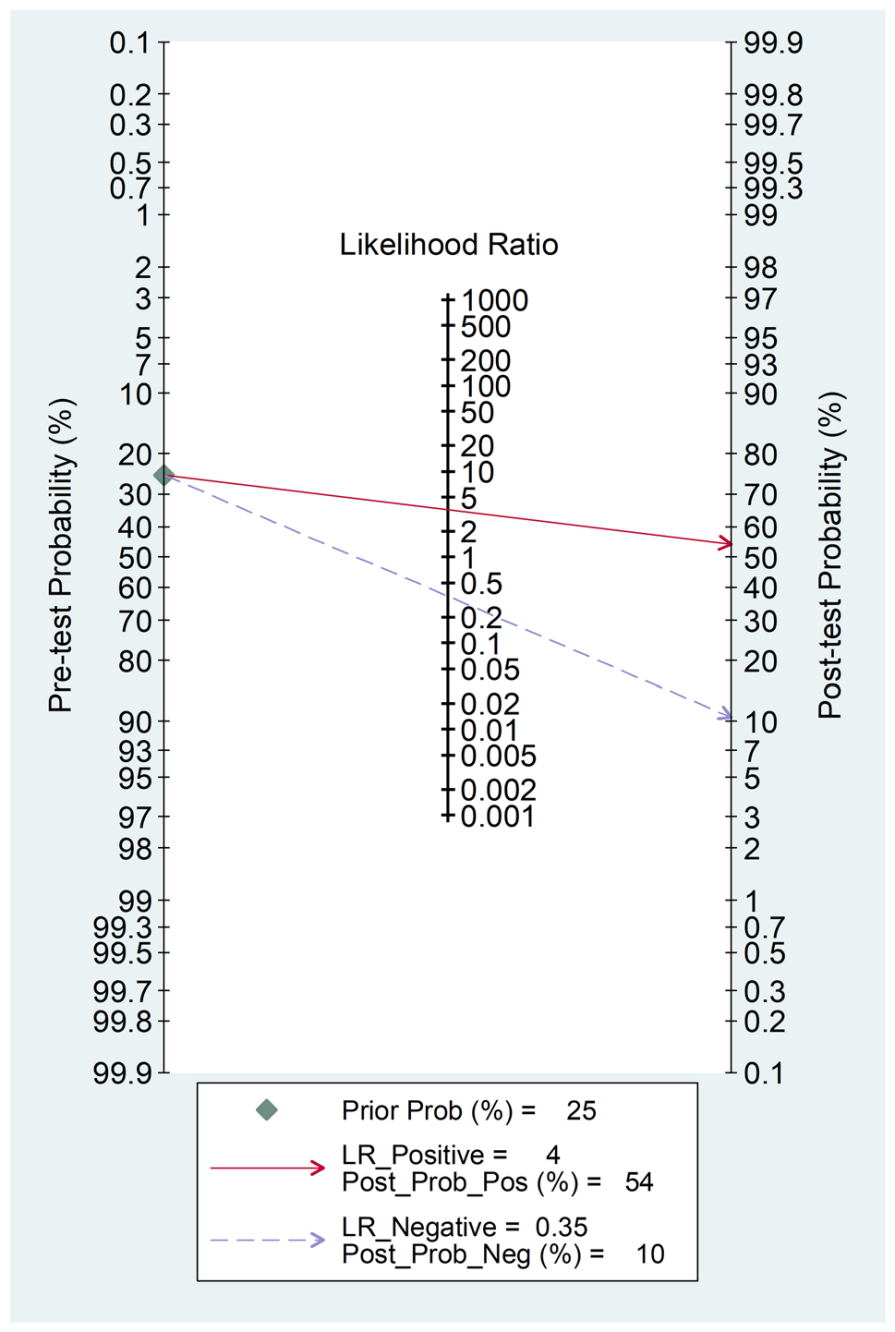
Fagan’s nomogram of serum amyloid A concentrations for the presence of rheumatic diseases

The pooled sensitivity and specificity were 0.72 (95% CI 0.63–0.79) and 0.80 (95% CI 0.68–0.88), respectively (Fig. 9). The SROC curve with 95% confidence region and prediction region showed an AUC value of 0.81 (95% CI 0.78–0.84), with the summary operating point at sensitivity of 0.72 and specificity of 0.80 (Fig. 10). The Fagan’s nomogram showed that, assuming a pre-test probability of RDs of 25%, the post-test probability was 54% in patients with relatively high SAA concentrations and 10% in those with relatively low SAA concentrations (Fig. 11).

### Serum amyloid A concentrations and disease activity

Disease activity was evaluated in eight studies investigating 500 RD patients (274 with active disease and 226 in remission) [41, 48, 56, 61, 68, 69, 71, 72] (Table 3). Six studies were conducted in Asia [41, 48, 61, 68, 69, 72], one in America [56], and one in Africa [71]. Three study investigated patients with TA [48, 61, 69], two with RA [56, 71], one with BD [41], one with AS [68], and one with gout [72].

The risk of bias was assessed as low in all studies except one, which exhibited moderate risk [41] (Supplementary Table 2).

**Fig. 12:**
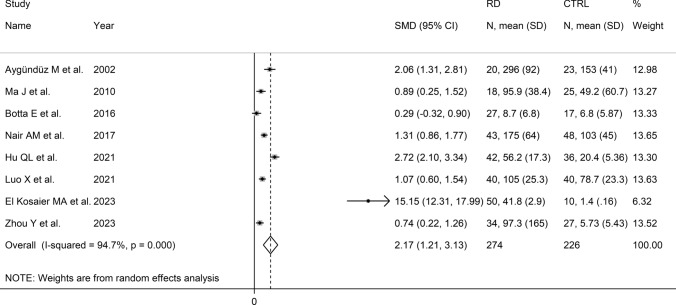
Forest plot of studies reporting serum amyloid A concentrations in patients with rheumatic diseases with active disease and patients in remission

**Fig. 13:**
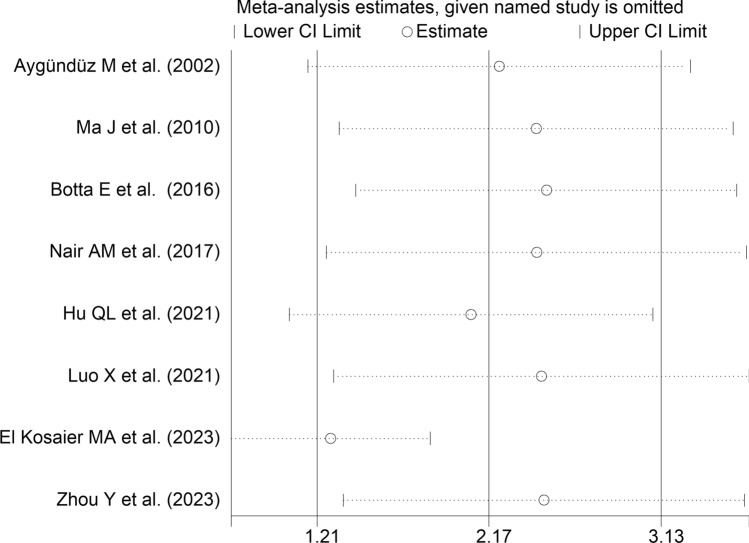
Sensitivity analysis of the association between serum amyloid A concentrations and the presence of active disease

The forest plot showed that SAA concentrations were significantly higher in RD patients with active disease when compared to those in remission (SMD = 2.17, 95% CI 1.21–3.13, *p* < 0.001; I^2^ = 94.7%, *p* < 0.001; Fig. 12). Sensitivity analysis showed the significant effect of one study on the corresponding pooled SMD values [71] (effect size ranged between 1.28 and 2.47) (Fig. 13). Removing this study reduced the pooled SMD which, however, remained significant (SMD = 1.28, 95% CI 0.73–1.84, *p* < 0.001; I^2^ = 85.3%, *p* < 0.001).

Assessment of publication bias, meta-regression, and subgroup analysis could not be conducted because of the insufficient number of studies.

The overall level of certainty was downgraded to very low after considering the low-moderate risk of bias in all studies (no change), the high and unexplained heterogeneity (downgrade one level), the lack of indirectness (no change), the large effect size (SMD = 2.17, upgrade one level) [73], and the lack of assessment of publication bias (downgrade one level).

## Discussion

This systematic review and meta-analysis has shown that SAA concentrations are significantly higher in patients with RDs when compared to healthy controls and in RD patients with active disease when compared to those in remission. In meta-regression and subgroup analysis, the effect size of the differences in SAA concentrations between RD patients and controls was not associated with and age, sample size, RD duration, CRP, ESR, total, HDL-, and LDL-cholesterol, and use of DMARDs or corticosteroids. By contrast, significant associations were observed with sex, body mass index, type of RD and study continent. In particular, there were significant differences vs. controls in studies of RA, SLE, FMF, TA and AS patients, but not in those in SSc or OA patients. Furthermore, studies conducted in Africa, unlike other continents, failed to report significant differences in SAA concentrations between RD patients and controls.

The lack of significant associations between the effect size and routinely used inflammatory biomarkers, i.e., CRP and ESR, suggests that the information provided by measuring SAA may complement, rather than duplicate, that provided by the CRP and ESR. Furthermore, the lack of association with disease duration suggests that the differences in SAA concentrations between RD patients and controls are likely to be manifest also in the early stages of the disease, potentially facilitating diagnosis and commencement of treatment. The reported associations between effect size and sex, indicating a relatively greater difference in SAA concentrations vs. controls in studies with a greater representation of male RD patients, represents an interesting finding as previous reports have shown similar SAA concentrations between males and females in healthy subjects [74, 75], and in patients with cancer [76]. Similarly, the significant and negative association observed between the effect size of the between-group differences in SAA concentrations and body mass index is at odds with previous reports which highlighted positive associations between SAA, body mass index, and obesity in non-RD populations [77, 78]. Future studies are required to confirm these findings and to investigate the pathophysiological and clinical significance of sex-related and body mass index-related differences in SAA concentrations in patients with RDs. The lack of significant differences in SAA concentrations vs. controls in patients with SSc or OA and in studies conducted in Africa needs to be interpreted with caution given the relatively small number of studies analysed (n = 2 for each of SSc, OA, and African patients).

Another important finding of our study was the good diagnostic performance of SAA concentrations for the overall presence of RDs, with pooled sensitivity, specificity, and AUC values of 0.72, 0.80, and 0.81, respectively. These figures compare favourably with those reported in studies investigating the diagnostic accuracy of CRP and ESR. For example, a prospective study assessing data from the Clinical Practice Research Datalink in UK primary care in 136,961 patients reported that the sensitivity and the specificity for any disease including infection, autoimmune disease, or cancer were 45.6 (95% CI 44.5–46.6) and 78.8 (96% CI 78.6–79.1) for CRP, and 42.0 (95% CI 40.8–43.2) and 78.6 (96% CI 78.3–78.9) for ESR [13]. Furthermore, the AUC value for autoimmune conditions was 0.71 (95% CI 0.70–0.72) for CRP and 0.71 (95% CI 0.69–0.72) for ESR whereas the AUC for combined CRP and ESR was marginally higher, 0.72 (95% CI 0.71–0.74), but still considerably below that observed for SAA in our study. The significant separation observed in Fagan’s nomogram, with more than doubling of the post- vs. pre-test probability (54% vs. 25%) in patients with relatively high SAA concentrations and more than halving (10% vs. 25%) in those with relatively low SAA concentrations further supports the promising role of SAA as a biomarker of RDs. These results, however, need to be corroborated by appropriately designed prospective studies conducted in different types of RDs to investigate whether the SAA can significantly improve the diagnosis and management of this group and complement the information provided by current recommendations, i.e., clinical evaluation, imaging studies, inflammatory biomarkers, and specific serological markers. Another critical issue requiring study is whether SAA provides added diagnostic value when measured before, during, or after measuring conventional inflammatory biomarkers such as CRP and ESR.

Strengths of our systematic review and meta-analysis include the comprehensive assessment of SAA concentrations in different types of RDs, the evaluation of diagnostic accuracy, and the significant between-group differences observed in studies conducted in most continents, which supports the generalizability of our findings. One significant limitation was the relatively limited evidence available in patients with specific RDs, particularly SSc, OA, PMR, gout, HSP, PsA, and SpA.

In conclusion, the results of our systematic review and meta-analysis suggest that SAA is a promising biomarker for the overall diagnosis of RDs and the presence of active disease. Further prospective studies should investigate whether the diagnostic information provided by SAA significantly complements that provided by clinical evaluation, imaging studies, and available biomarkers, consequently enhancing the assessment and management of patients with RDs.

## Supplementary Information

Below is the link to the electronic supplementary material.

Supplementary file1 (DOCX 35 KB)

Supplementary file2 (DOCX 63 KB)

## Data Availability

The data that support the findings of this systematic review and meta-analysis are available from AZ upon reasonable request.
