# Supplementary material for: The potential role of serum amyloid A as biomarker of rheumatic diseases: a systematic review and meta-analysis
Source: Clin Exp Med. 2024 Jun 29;24(1):141. doi: 10.1007/s10238-024-01413-0 (PMC11217051; doi:10.1007/s10238-024-01413-0)
Supplement: Supplementary file 2 — Supplementary file2 (DOCX 63 KB) [file 10238_2024_1413_MOESM2_ESM.docx]

**Supplementary Table 2.** Assessment of the risk of bias using the Joanna Briggs Institute critical appraisal checklist.

| **Study** | **Were inclusion criteria clearly defined?** | **Were the subjects and the setting described in detail?** | **Was the exposure measured in a reliable way?** | **Were standard criteria used to assess the condition?** | **Were confounding factors identified?** | **Were strategies to deal with confounding factors stated?** | **Were the outcomes measured in a reliable way?** | **Was appropriate statistical analysis used?** | **Risk of bias** |
| --- | --- | --- | --- | --- | --- | --- | --- | --- | --- |
| Aygündüz M et al. [1] | No | Yes | Yes | Yes | No | No | Yes | Yes | Moderate |
| Tanimoto N et al. [2] | No | Yes | Yes | Yes | Yes | Yes | Yes | Yes | Low |
| Wong M et al. [3] | Yes | Yes | Yes | Yes | Yes | Yes | Yes | Yes | Low |
| Jung SY et al. [4] | Yes | Yes | Yes | Yes | No | No | Yes | Yes | Low |
| Karadag O et al. [5] | Yes | Yes | Yes | Yes | No | No | Yes | Yes | Low |
| Sato H et al. [6] | No | Yes | Yes | Yes | Yes | Yes | Yes | Yes | Low |
| Rho YH et al. [7] | No | Yes | Yes | Yes | Yes | Yes | Yes | Yes | Low |
| Ma J et al. [8] | Yes | Yes | Yes | Yes | No | No | Yes | Yes | Low |
| Rooney T et al. [9] | Yes | Yes | Yes | Yes | Yes | Yes | Yes | Yes | Low |
| He X et al. [10] | Yes | Yes | Yes | Yes | Yes | Yes | Yes | Yes | Low |
| Londono J et al. [11] | No | Yes | Yes | Yes | No | No | Yes | Yes | Moderate |
| de Seny D et al. [12] | Yes | Yes | Yes | Yes | No | No | Yes | Yes | Low |
| Giese A et al. [13] | No | Yes | Yes | Yes | No | No | Yes | Yes | Moderate |
| Lakota K et al. [14] | No | Yes | Yes | Yes | No | No | Yes | Yes | Moderate |
| Shen C et al. [15] | No | Yes | Yes | Yes | No | No | Yes | Yes | Moderate |
| Botta E et al. [16] | Yes | Yes | Yes | Yes | Yes | Yes | Yes | Yes | Low |
| Ciftci S et al. [17] | Yes | Yes | Yes | Yes | No | No | Yes | Yes | Low |
| Gaál K et al. [18] | Yes | Yes | Yes | Yes | Yes | Yes | Yes | Yes | Low |
| Uslu AU et al. [19] | Yes | Yes | Yes | Yes | No | No | Yes | Yes | Low |
| Fentoglu O et al. [20] | Yes | Yes | Yes | Yes | No | No | Yes | Yes | Low |
| Nair AM et al. [21] | Yes | Yes | Yes | Yes | No | No | Yes | Yes | Low |
| Lis-Swiety A et al. [22] | Yes | Yes | Yes | Yes | No | No | Yes | Yes | Low |
| Bezuidenhout JA et al. [23] | Yes | Yes | Yes | Yes | Yes | Yes | Yes | Yes | Low |
| van Sleen Y et al. [24] | No | Yes | Yes | Yes | No | No | Yes | Yes | Moderate |
| Yuan X et al. [25] | Yes | Yes | Yes | Yes | No | No | Yes | Yes | Low |
| Can Sandikci S [26] | Yes | Yes | Yes | Yes | No | No | Yes | Yes | Low |
| Ciregia F et al. [27] | No | Yes | Yes | Yes | No | No | Yes | Yes | Moderate |
| Hu QL et al. [28] | Yes | Yes | Yes | Yes | No | No | Yes | Yes | Low |
| Luo X et al. [29] | Yes | Yes | Yes | Yes | No | No | Yes | Yes | Low |
| Sweet K et al. [30] | Yes | Yes | Yes | Yes | No | No | Yes | Yes | Low |
| El Kosaier MA et al. [31] | Yes | Yes | Yes | Yes | No | No | Yes | Yes | Low |
| Zhou Y et al. [32] | Yes | Yes | Yes | Yes | Yes | Yes | Yes | Yes | Low |
